# Tailoring the Edge Sites of 2D Pd Nanostructures with Different Fractal Dimensions for Enhanced Electrocatalytic Performance

**DOI:** 10.1002/advs.201800430

**Published:** 2018-06-10

**Authors:** Yucong Yan, Xiao Li, Min Tang, Hao Zhong, Jingbo Huang, Ting Bian, Yi Jiang, Yu Han, Hui Zhang, Deren Yang

**Affiliations:** ^1^ State Key Laboratory of Silicon Materials and School of Materials Science & Engineering Zhejiang University Hangzhou 310027 China; ^2^ Department of Mathematics Zhejiang University Hangzhou 310027 China; ^3^ School of Energy and Power Engineering Jiangsu University of Science and Technology Zhenjiang 212003 China

**Keywords:** edge sites, electrocatalysis, fractal dimension, nanosheets, palladium

## Abstract

The important role of edge sites in atomically thin 2D materials serving as catalysts is already of concerned in plenty of material systems and catalytic reactions, whereas comprehensive study of the edge sites in 2D noble‐metal nanocatalysts is still lacking. Herein, for the first time, a controllable etching approach to tailor the fractal dimensions and edge sites of Pd nanosheets is developed and the edge sites in these 2D nanostructures from both structural and chemical aspects are investigated. The as‐tailored 2D Pd nanostructures with extra edge sites exhibit substantially enhanced electrocatalytic performance for the formic acid oxidation reaction (FAOR). Moreover, careful analysis of the results from electrocatalytic measurements reveals that the specific activities for the edge sites in the 2D nanostructures far exceed the activities for the low‐index planes of Pd and even dominate the overall activity exhibited by the 2D noble‐metal catalysts.

At the forefront of catalysis there exists a research endeavor centered around 2D nanomaterials, which present great promise as catalysts due to their unique structural and electronic properties.^1^, ^2^ As a special category of 2D materials, ultrathin nanosheets composed of noble metals, which have irreplaceable roles in various catalytic applications, have recently been the topic of increasing research interests.^3^, ^4^, ^5^, ^6^ These 2D nanostructures show a high specific surface area as well as high ratio of unsaturated sites on the surface, which can maximize utilization of the noble‐metal atoms, and thus significantly ameliorate catalytic performance.^7^, ^8^, ^9^ For instance, atomically thick Rh nanosheets fabricated by Li and co‐workers showed remarkable enhancement in both activity and selectivity as a catalyst for hydrogenation and hydroformylation reactions compared to Rh nanoparticles and commercial Rh/C.^10^ Xiao and co‐workers synthesized Au nanosheets enclosed within {001} basal planes through an anion‐exchange process in layered double hydroxides, which showed excellent catalytic performance for selective oxidation of ethylbenzene and toluene with molecular oxygen as the oxidant.^11^ Notwithstanding the superiority presented in the design of the 2D nanostructures, some inherent merits of the 2D noble‐metal materials are restricted. This fact is partially a result of the irregular (re)stacking of 2D nanocrystals driven by high surface energy, which can severely shrink the accessible surface area.^12^, ^13^ To address this issue, self‐supported nanoarchitectures with altered dimensions have been constructed using 2D materials.^14^, ^15^, ^16^ Huang and co‐workers demonstrated that three‐dimensional (3D) superstructures composed of ultrathin Ir nanosheets enabled fabrication of one of the best catalysts for the oxygen evolution reaction in both alkaline and acid conditions.^17^ Moreover, the basal planes and dominant exposed facets of freestanding 2D noble‐metal nanocrystals can rarely be adjusted while the surface structure of such nanocrystals generally plays a key role for specific catalytic reactions.^18^, ^19^ To overcome this predicament, 2D nanostructures with abundant defects, such as edges for instance, as extra active sites are prepared to compensate for the low activity of relatively inert facets.^20^ Even though this strategy has been widely employed in plenty of other 2D materials, such as oxides, sulfides, and carbon, unfortunately, very limited successes has yet been demonstrated for 2D noble‐metal nanocrystals containing excess edges or other defective sites.^21^, ^22^, ^23^, ^24^, ^25^, ^26^, ^27^, ^28^, ^29^ Moreover, comprehensively investigating the structural and catalytic features of the edge sites in 2D noble‐metal nanocrystals remains an important open challenge. Herein, we report our research effort in developing a refined synthetic approach for preparing low‐dimensional Pd nanocrystals with tuned edge sites and constructing a structure–property relationship for use of these nanostructures as electrocatalysts. A series of Pd nanostructures with rationally tailored dimensions and edge sites including Pd porous nanosheets (PNSs), Pd nanobelts (NBs), and Pd 2D nanoframes (2D NFs), were synthesized through controllable etching of as‐prepared Pd fractal nanosheets (FNSs). The geometric and physical features of these nanostructures were investigated through morphology and structure characterization along with mathematical modeling, with different types of typical edge sites identified in the 2D nanostructures. In addition, formic acid oxidation reaction (FAOR) was chosen as the model reaction to evaluate the catalytic performance. Following an analysis based on the results of electrochemical measurements, one comes to some unexpected conclusions: 1) the edge sites for the Pd 2D NFs and Pd FNSs show an ≈25 times higher specific activity for FAOR compared to the Pd {111} facets, 2) the atoms at the edge sites, which only occupy a tiny proportion (less than 5%) of the sites in the 2D Pd nanocrystals, can practically lead to tremendous contribution (up to 90%) to the overall catalytic activity.

In a typical synthesis of Pd PNSs, Pd FNSs were first prepared as seeds and then dispersed and heated in an *N*, *N*‐dimethylformamide (DMF) solution of palladium(II) acetylacetonate (Pd(acac)_2_), hexadecyltrimethylammonium bromide (CTAB), ascorbic acid (AA), and sodium acetate (NaAc) at 60 °C in air. The synthesis of Pd NBs was conducted without the addition of NaAc, while Pd 2D NFs were synthesized at 80 °C (see the Supporting Information for details). Representative transmission electron microscopy (TEM) and high‐angle annular dark‐field scanning transmission electron microscopy (HAADF‐STEM) images (Figures S1 and S2, Supporting Information) of the products indicate good morphology and size uniformity, while typical TEM images (**Figure** **1**) of individual planar nanocrystals taken at higher magnification reveal more details for the morphology. As shown in Figure 1a, the seeds for the Pd FNSs present a hexagonal shape with several fractal subunits overlapped around nodes at the periphery. These Pd FNSs are further demonstrated to be single crystalline according to the results obtained from the selected area electron diffraction (SAED) pattern (Figure S3, Supporting Information) of an individual Pd FNS. After a selective etching/deposition process, this sheet‐like structure was undermined in different degrees and transformed into Pd PNSs, NBs, and 2D NFs (Figure 1b–d) with increased average thickness (Figures S4–S6, Supporting Information). The average thickness of the Pd 2D nanostructures was measured based on TEM images (Figures S4 and S5, Supporting Information) taken for nanostructures that were of vertically assembled or vertically attached onto the carbon nanotubes. We also utilized atomic force microscopy (AFM) to measure the thickness of the Pd FNSs (Figure S6, Supporting Information). Except for a few narrow steps in the AFM height profiles (Figure S6b,c, Supporting Information), which agree well with the thickness obtained from the TEM images (≈1.2 nm), most of the results show a higher thickness due to the adsorbed poly(vinylpyrrolidone (PVP). As for the Pd PNSs, the average thick‐ness increased by 0.3 nm compared to the Pd FNSs, with the planar surface found to be riddled with small round holes with mostly curved edges (Figure 1b). Pd NBs present a belt‐like quasi‐1D structure with distorted shapes (Figure 1c) and notably increased average thickness (≈3.9 nm), which can link to each other as a network (Figures S1c and S2c, Supporting Information). By contrast, Pd 2D NFs consist of fine girders with an average thickness of ≈2 nm and high density of cavities with inside edges that are straight and parallel to the outside periphery.

**Figure 1:**
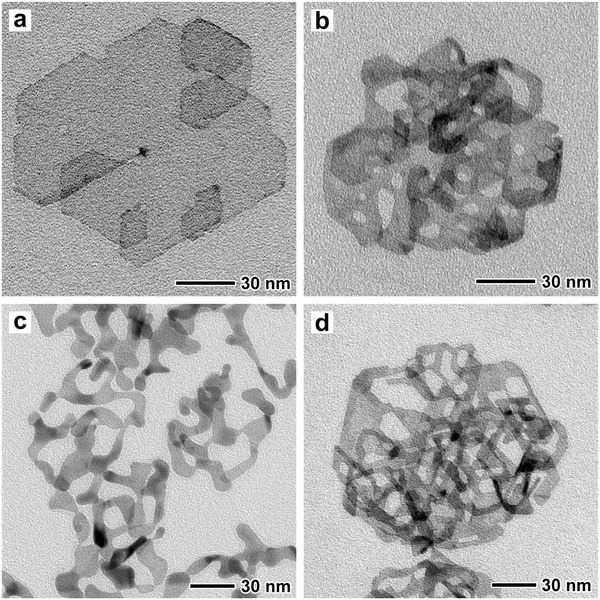
Representative TEM images for a) Pd FNSs, b) Pd PNSs, c) Pd NBs, and d) Pd 2D NFs.

Moreover, aberration‐corrected HAADF‐STEM was used to investigate the structural features for the edge sites in the nanocrystals on the atomic scale (**Figure** **2**; Figures S7–S9, Supporting Information). The HAADF‐STEM images taken for flat nanocrystals indicate a lattice spacing of 0.14 and 0.23 nm, which can be indexed to the {220} and {111} planes of Pd. Remarkably, two distinct forms of edges can be identified from typical HAADF‐STEM images (Figure 2) focused on a hole in one Pd PNS and a cavity in one Pd 2D NF, respectively. Compared with the crystal orientation that is determined by the lattices, the edges in the Pd 2D NFs are orientated along the <110> direction, while the edges for the holes in the Pd PNSs show irregular shapes terminated by atoms with an abundance of dangling bonds. According to the gradually fading contrast across the edges shown in the images and corresponding contrast profiles (Figure 2; Figure S7, Supporting Information), the edges in these Pd nanostructures differ significantly in their width as well as decreased thickness, revealing various slopes or shapes of these edges, which can be quantitatively described by a shape factor γ (for more details see the Supporting Information). Comparatively speaking, the edges in the Pd PNSs are the gentlest for the biggest value of γ (Table S1, Supporting Information), implying massive low‐coordinated atoms. The edges of the Pd FNSs and 2D NFs are relatively abrupt, with values for γ very close to each other, indicating that similar atomic structures exist for these edges. In addition, the edges of the Pd NBs are steepest with a dramatically increased thickness and plenty of surficial defects such as terraces and steps can be found (Figures S8b and S9, Supporting Information). Other characteristic statistical parameters which describe the shapes and edge sites in the Pd nanostructures such as the fractal dimensions and percentage of atoms at the edge sites, are also summarized in Table S1 in the Supporting Information.

**Figure 2:**
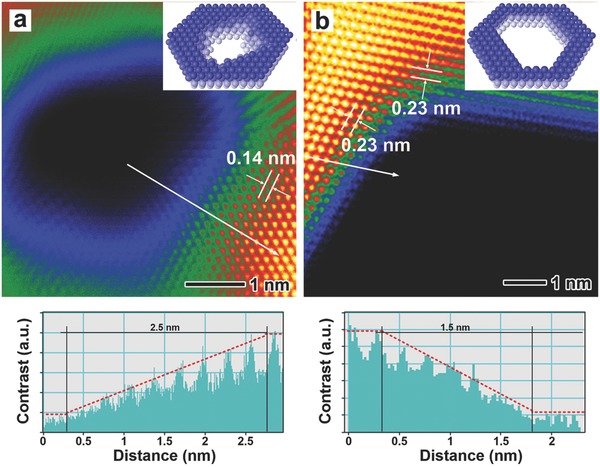
Atomic resolution HAADF‐STEM images and contrast profiles measured along the arrowed lines of the inner edges for a) Pd PNSs and b) Pd 2D NFs. False color was applied in the STEM images to improve the contrast. The insets show the corresponding atomic models.

The physicochemical features of these Pd nanostructures were further investigated using X‐ray photoelectron spectroscopy (XPS) and electron spin resonance (EPR) techniques. As shown in **Figure** **3** and Figure S10 (Supporting Information), the 3d peaks in the XPS spectra for Pd PNS, NBs, and 2D NFs are broadened to different degrees relative to the Pd FNSs, which reveals an increase in the percentage of atoms at the edge sides apart from the planar planes. From the point of view of the peak position, a notable downward shift of ≈0.4 eV for the main peaks and the appearance of a peculiar red‐shifted fitting peak can be observed from in the 3d spectra for the Pd PNSs and NBs relative to the Pd FNSs, which can be attributed to the surface core‐level shifts arising from the low‐coordinated atoms at surficial defect sites.^30^ Conversely, the values for the binding energy shown in Pd 2D NFs are in accordance with the peaks identified for the Pd FNSs that are basically composed of the fitting peaks of Pd^0^, Pd^II^, and PdO, implying a similarity for the edge sites on these two nanostructures not only in a geometrical sense but a chemical sense. In addition, some small fitting peaks (Figure S10, Supporting Information) with substantially increased binding energy observed in the XPS spectra of Pd PNSs, NBs, and 2D NFs can be attributed to the absorption of Br^−^ and oxygen group, which are in introduced in the etching process. Considering that the 3d orbits for Pd^0^ are full without any single electron, EPR signals can be used to reflect the electronic states of the defective sites in the Pd nanocrystals.^31^, ^32^ The EPR spectrum (Figure 3b; Figure S11, Supporting Information) evidence the aforementioned similarity and diversity reflected in the Pd nanostructures both in the aspects of signal forms and positions. The waveforms shown by the second harmonic EPR signals for the Pd FNSs and 2D NFs agree with the typical form of the axisymmetric crystals, while the sample types exhibited by the waveforms for the Pd PNSs and NBs are anisotropic but unsymmetrical.^33^, ^34^ In addition, the main peaks for the Pd PNSs and NBs are evidently wide with a lower *g*‐value of ≈4.0, indicating strong spin–orbit coupling in the atoms at the defective sites. For the signals measured for the Pd FNSs and 2D NFs, the relatively narrow waveforms and raised *g*‐value (≈4.2) can be attributed to the result of both spin–orbit coupling and a symmetrical crystal field.^34^ Taken together, the straightly aligned edge sites on the Pd FNSs and 2D NFs share similar structural and physicochemical features while Pd PNSs and NBs show more particular characteristics.

**Figure 3:**
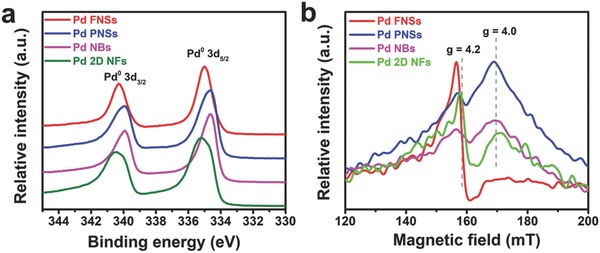
Characterizations of the chemical states of different Pd nanostructures: a) the Pd 3d XPS spectra, b) EPR spectra for the second harmonic resonance at 1.8 K.

To understand the formation mechanism for these distinct nanostructures, the roles of the various reagents and the reactive process at different time points are investigated. According to the results of controlled trials (Figure S12, Supporting Information) in which the additive amounts of the specific chemicals were changed, the etching process was triggered by the joint effect of O_2_, and Br^−^ as reported in the literatures (Figure S12a, Supporting Information),^35^, ^36^ while the proton or H^+^ is also crucial to accelerate this etching process (Figure S12b,c, Supporting Information). It is worth noting that AA plays two different roles as reductant and acid, whose weight can be adjusted with the addition of NaAc as weak base (Figure S12d, Supporting Information). As shown in Figure S13 in the Supporting Information, the results from the time‐dependent experiments reflect more details during the morphology evolution from a kinetic aspect. At the initial stage of the reactions (*t* = 15 min), tiny pinholes began to appear inside the Pd FNSs. As the reaction time increased (*t* = 30 and 60 min), these pinholes inside the nanosheets proliferated, expanded, and merged with different rates while the remaining parts became thickened at the same time. As a result, these similar reaction processes with a varying kinetic equilibrium between etching, deposition, and surface diffusion led to diverse final products. For the Pd PNSs, the etching process was constrained inside while the outer periphery composed of newly deposited atoms was protected by the increased thickness. Without the addition of NaAc, the etching process sped up, whereas the reduction and deposition of Pd atoms slowed down, cutting apart the nanosheets into nanobelts. In the synthesis of Pd 2D NFs, the rate of surface diffusion became significantly elevated under higher reaction temperature, leading to the formation of cavities with regular shapes and straight edges.

Except for use as catalysts in a variety of organic heterogeneous reactions, Pd nanomaterials are believed to be promising electrocatalysts for electrode reactions in fuel cells, especially FAOR. Therefore, we evaluated the catalytic performance of supportless 2D Pd nanocrystals for FAOR in acid medium benchmarked using commercial Pd black. The electrochemically active surface areas (ECSAs) for these catalysts were calculated from the carbon monoxide oxidation charge in the CO‐stripping curves (Figure S14, Supporting Information). As observed in Table S2 in the Supporting Information, the ECSAs for these 2D Pd nanostructures show a positive correlation with the corresponding fractal dimension and decrease with thickness. **Figure** **4**a and Figure S15a (Supporting Information )compare the electrochemical specific area as well as mass specific current densities obtained from the peaks and cyclic voltammograms (CV) curves measured for these four catalysts and commercial Pd black for FAOR carried out in an aqueous solution containing 0.5 m H_2_SO_4_ and 0.5 m formic acid. As indicated in Figure 4a, the specific activity of the original Pd FNSs is even lower than commercial Pd black due to the dominant percentage of surface atoms in the {111} planes of the Pd FNSs, which are believed to be less active crystal planes for FAOR.^37^ Compared with Pd FNSs and Pd black, Pd PNSs, NBs, and 2D NFs exhibit enhanced specific activities due to the increased ratio of atoms at the edge sites. However, the ratio of the surface atoms at the edge sites and specific activities of these Pd 2D catalysts are not linearly correlated, suggesting that the specific activity of the Pd 2D catalysts depends upon not only the percentages but the types of edge sites. The mass activities for the Pd FNSs, PNSs, NBs, and 2D NFs are 2.54, 3.21, 1.44, and 3.05 times higher than that for commercial Pd black, since the larger ECSAs of the low dimensional nanostructures make up for the low activity of the {111} planes. Moreover, the durability of these catalysts toward FAOR was investigated by testing over 500 more cycles in a mixed solution of formic acid and H_2_SO_4_. As shown in Figure 4b and Figure S15b (Supporting Information), after the durability test commercial Pd black eliminate most of its activity while Pd FNSs, PNSs, NBs, and 2D NFs retain 33.1%, 11.5%, 61.8%, and 46.0% of their activities, respectively. According to the TEM images (Figures S16 and S17, Supporting Information) of the catalysts taken after the durability test, the destruction and agglomeration of nanostructures is responsible for the decline in the catalytic performance. In particular, fragmentation can be found for Pd FNSs and 2D NFs while Pd NBs remains almost intact because of the increased thickness. Interestingly, plenty of ultrasmall Pd nanoparticles can be observed at the edges of the Pd PNSs, indicating a process of dissolution and recrystallization. This peculiar phenomenon, together with the relatively poor durability among the 2D Pd nanostructures, shows that the edge sites in Pd PNSs are more structurally unstable compared with other samples.

**Figure 4:**
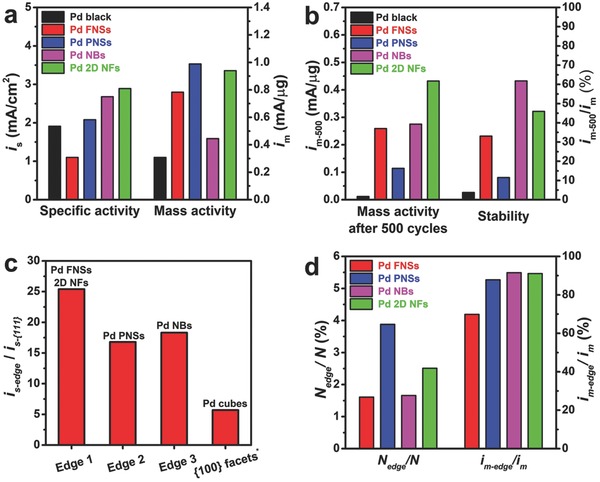
a) Specific and mass activities for commercial Pd black, Pd FNSs, PNSs, NBs, and 2D NFs at the peak position of the forward curve in FAOR measurements. b) Mass activities and relative stabilities measured after 500 cycles in FAOR tests. c) Average specific activities for different types of edges and Pd {100} facets relative to the Pd {111} facets.^38^ d) The atomic ratio of edge atoms relative to total atoms and the percentage of activity contributed by the edge sites.

To specifically investigate the catalytic properties of the edge sites as a more meaningful indicator, the overall electrocatalytic currents for 2D nanostructures are regarded as a linear combination of the currents for the {111} planes and different types of edges sites. Through mathematical modeling based on the TEM images (for more details in Supporting Information), the percentage of edge atoms in each nanostructure, namely the coefficients in the linear combination, can be derived (Table S1, Supporting Information). According to the aforementioned characterization, the similarity between the edge sites for the Pd FNSs and 2D NFs were corroborated both structurally and physically. Consequently, it is well founded to suppose that the catalytic features of the edge sites in these two nanostructures are similar. Given this approximation, it is possible to solve the matrix for those linear combinations of currents. As shown by the solution in Figure 4c, the three types of edge sites present in the Pd FNSs as well as the 2D NFs (Edge 1), Pd PNSs (Edge 2), and Pd NBs (Edge 3) exhibit ultrahigh specific activities, which are 25.4, 16.8, and 18.3 times higher than the specific activity of the {111} planes respectively. Taking the activity and durability together, the form of the edges with a straight alignment along the <110> direction shows a better performance for FAOR compared to the form of the edges with irregular outlines. It is notable that the enhancements in the specific activities shown by the edge sites in the 2D Pd nanostructures are also remarkably higher than the specific activity of the Pd {100} planes (≈5.7 times higher than the value for the {111} planes reported in literature),^38^ which are considered as the most active low‐index planes of Pd for FAOR. Moreover, when the solution of the matrix is substituted into the linear combination, the percentage of the catalytic activity contributed by the edge sites can be derived (Figure 4d). The data show that the majority of the activity due to the 2D Pd catalysts (at least 69.9% and up to 91.5%) is contributed by the atoms at the edge sites which account for only a tiny proportion (≈1.6% to 3.9%) of atoms in the nanocrystals.

In summary, we successfully synthesized a series of Pd 2D nanostructures with tailored fractal dimensions and edges via controllable etching, and systematically investigated the structural and chemical features for the edge sites as special catalytic centers. With the assistance of mathematic modeling, we found that the edge sites in these 2D Pd nanostructures exhibited the specific activities for FAOR that far exceeded the performance of Pd low‐index planes. Furthermore, this study demonstrated that the greatest proportion of the catalytic activity exhibited by 2D noble metal nanocrystals can be contributed by the edge sites, whose contribution might have been previously underestimated. We believe that the present work can shed light on the structure–property relationship for 2D noble‐metal nanomaterials and provide a fresh impetus for the rational design of defect‐rich nanocatalysts with maximized utilization efficiency of atoms.

## Experimental Section

*Synthesis of Pd FNSs*: First, Pd(acac)_2_ (16 mg), PVP (30 mg), and oxalic acid (60 mg) were dissolved in DMF (10 mL) and stirred for 1 h at room temperature. The homogeneous solution was then transferred into a 20 mL glass vial and W(CO)_6_ (100 mg) was added into the vial. These processes were carried out in argon atmosphere. The sealed vial was heated at 60 °C under magnet stirring for 3 h before cooling down to room temperature. The resulting dark blue solution was preserved in argon atmosphere for further use as seeds.

*Synthesis of Pd PNSs, NBs, and 2D NFs*: PVP (50 mg), AA (50 mg), CTAB (60 mg), and NaAc (2 mg) was dissolved in of DMF (8 mL) in a 20 mL capped glass vial. Then 3 mL of dark blue solution containing Pd FNSs was separated by centrifugation with acetone, and redispersed in the abovementioned reaction solution. Subsequently, DMF solution (2 mL) containing Pd(acac)_2_ (2 mg) dissolved was injected into the abovementioned mixture solution. The resulting mixture solution was heated at 60 °C for 3 h and then cooled to room temperature. The final product was collected by centrifugation followed by washing with ethanol three times. In the synthesis of Pd NBs, NaAc was not added under otherwise identical experimental conditions. In the synthesis of Pd 2D NFs, the reaction was conducted at 80 °C while all other experimental conditions remained the same.

*Electrochemical Measurements*: A total of 10 µg Pd FNSs, Pd PNSs, Pd NBs, and Pd 2D NFs catalysts and 5 µg commercial Pd black were loaded onto working electrodes. Before electrochemical measurement, all five catalysts were electrochemically cleaned through a CV process in HClO_4_ (0.1 m) solution for decades of cycles. The metal loading on the glass carbon electrodes (GCE) for all samples was 10 µg. The ECSA was determined by integrating the carbon monoxide oxidation charge via CO stripping measurements. A scan rate of 50 mV s^−1^ was used for the CV measurement. For CO‐stripping measurement, the GCE with catalysts loaded was first immersed in 0.1 m HClO_4_ solution with CO bubbling for 15 min and then in argon saturated HClO_4_ solution (0.1 m) to conduct the stripping voltammograms. The FAOR was conducted in a mixture solution containing H_2_SO_4_ (0.5 m) and formic acid (0.5 m) at a scan rate of 50 mV s^−1^. The durability tests were carried out under the same condition as the FAOR tests for an additional 500 cycles.

## Conflict of Interest

The authors declare no conflict of interest.

## Supporting information

Supplementary
