# Supplementary material for: Tailoring the Edge Sites of 2D Pd Nanostructures with Different Fractal Dimensions for Enhanced Electrocatalytic Performance
Source: Adv Sci (Weinh). 2018 Jun 10;5(8):1800430. doi: 10.1002/advs.201800430 (PMC6096982; doi:10.1002/advs.201800430)
Supplement: Supplementary file 1 — Supplementary [file ADVS-5-1800430-s001.pdf]

Copyright WILEY-VCH Verlag GmbH & Co. KGaA, 69469 Weinheim, Germany, 2018.

ADVANCED SCIENCE
Open Access

Supporting Information

for *Adv. Sci.,* DOI: 10.1002/advs.201800430

Tailoring the Edge Sites of 2D Pd Nanostructures with Different Fractal Dimensions for Enhanced Electrocatalytic Performance

Yucong Yan, Xiao Li, Min Tang, Hao Zhong, Jingbo Huang, Ting Bian, Yi Jiang, Yu Han, Hui Zhang, and Deren Yang**

Supporting Information

Tailoring the Edge Sites of Two-dimensional Pd Nanostructures with Different Fractal Dimensions for Enhanced Electrocatalytic Performance

Yucong Yan, Xiao Li, Min Tang, Hao Zhong, Wei Tong, Jingbo Huang, Ting Bian, Yi Jiang, Yu Han, Hui Zhang* and Deren Yang*

DOI:

Contents of Supporting Information

Experimental Procedures
Modeling and Calculation (including Scheme S1 and S2)
Supporting Tables S1-S2
Supporting Figures S1-S17
References

Experimental Procedures

Chemical and Materials.

Palladium(II) acetylacetonate (Pd(acac)$_2$, 99%), poly(vinylpyrrolidone) (PVP, MW ≈ 29000), oxalic acid, hexadecyltrimethylammonium bromide (CTAB), ascorbic acid (AA), sodium acetate (NaAc), tungsten hexacarbonyl (W(CO)$_6$), formic acid and commercial Pd black were all purchased from Sigma Aldrich. N,N-Dimethylformamide (DMF), sulfuric acid (H_2SO_4), ethanol and acetone were purchased from Sinopharm Chemical Reagent. Deionized water (18.25 MΩ·cm) was used for all experiments. All syntheses were carried out in a glass vial (20 mL).

Morphological and structural characterizations.

Transmission electron microscopy (TEM) images of the obtained samples were taken using a HITACHI HT-7700 microscope operated at 100 kV. High-resolution transmission electron microscopy (HRTEM) was performed using a FEI Tecnai F30 G2 microscope operated at 300 kV. High-angle annular dark-field scanning TEM (HAADF-STEM) was taken on a FEI Titan Chemi-STEM equipped with a probe-corrector. This microscope was operated at 200 kV with a probe current of 50 pA and a convergent angle of 21.4 mrads for illumination. X-ray photoelectron spectrometer (XPS) was performed on ESCALAB 250Xi (Thermo, U.K). Electron spin resonance (EPR) tests were performed using a Bruker EMX plus on the X wave band at 1.8 K.

Details in Electrochemical Measurements.

A three-electrode cell was used to take the electrochemical measurement with a CHI760E electrochemical analyzer (CH Instrument, Shanghai). The working electrode was a glassy-carbon rotating disk electrode (GCE) (diameter: 5 mm and area: 0.196 cm^2) from Pine Instruments. A platinum wire with the length of 5 cm was used as the counter electrode and a reversible hydrogen electrode (RHE) which was calibrated with H_2 oxidation/evolution on a Pt polycrystalline RDE electrode was used as the reference electrode. The concentration of catalyst inks was determined using inductively coupled plasma atomic emission spectrometry (ICP-AES, IRIS Intrepid II XSP, TJA Co., USA). To prepare the working electrode, water dispersions containing 10 μg of Pd FNSs, Pd PNSs, Pd NBs and Pd 2D NFs catalysts were dropped on the GCE. After the dispersion was dried, Nafion solution (1 μL, 0.05%) was deposited on the working electrode. To prepare the inks of the reference catalysts, 5 mg of commercial Pd black (Sigma Aldrich) was dispersed in a mixed solution (5 mL) containing DI water,

isopropanol, 5% Nafion ethanol solution in the volume ratio of 3:1.975:0.025. After that, the ink was added onto the GCE by a pipette and dried in flowing air.

Methods of model construction and calculation

The calculation of fractal dimension of the Pd nanostructures.

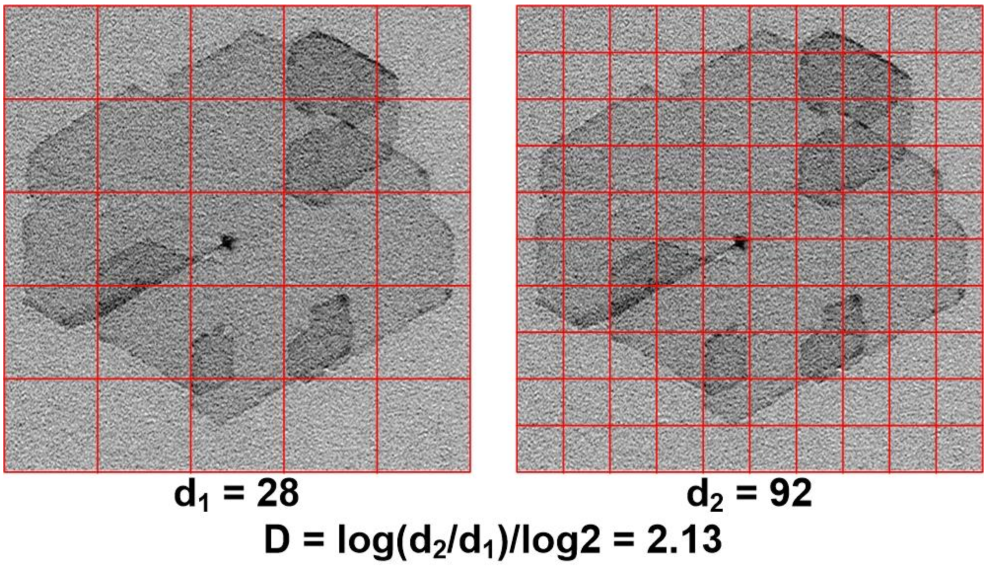

Scheme S1. Scheme of the calculation of D using a box-counting method.

A simple box-counting method could be used to calculate the fractal dimension (or Hausdorff dimension) [1-3] of different Pd nanostructures. As shown in Scheme S1, a TEM image of a Pd FNS was put into grids with different density, and the number of unit squares with Pd FNS (d_i) filled was counted. In the zones involved with several layers, repeat counting was utilized. The fractal dimension (D) can be expressed with the relationship between the ratio of d_i and the ratio of the densities of grids. All TEM images used had identical side length (150 nm), and the densities of grids were 5×5 and 10×10. The same calculation was conducted for dozens of samples to obtain average results.

The calculation of the average specific activities of different edge sites.

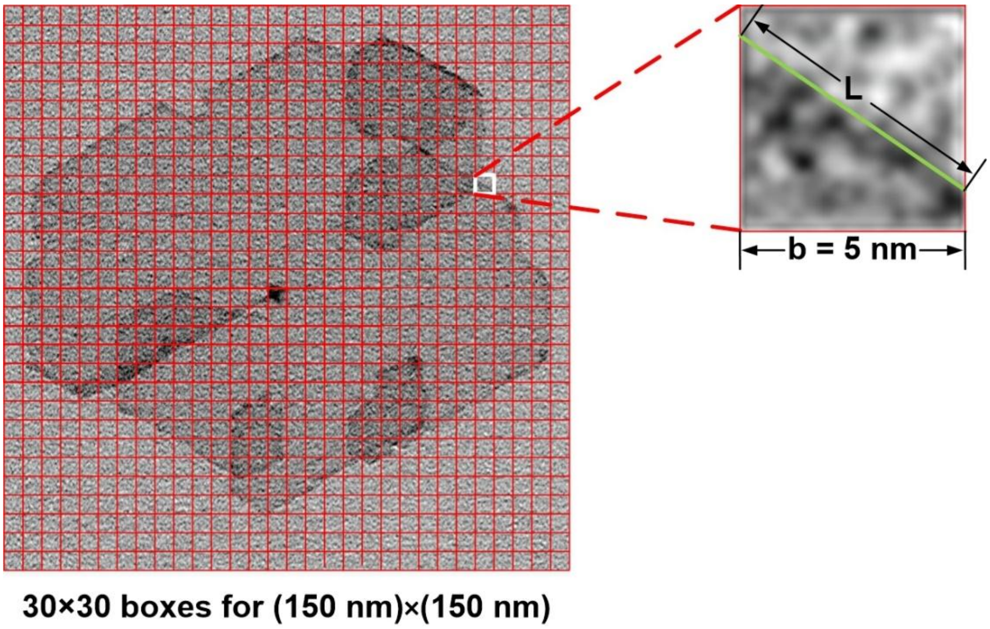

Scheme S2. Scheme of the calculation of planar surface and edge areas of the Pd nanostructures.

In order to obtain the average area specific activities of the edge sites in the Pd nanostructures, the percentage of Pd atoms in different structural states have to be derived first. We solved this problem via box-counting again. Presented as Scheme S2, the TEM image of a horizontally lied Pd nanocrystal was cut up into lots of unit boxes with a fine grid. The side length of the small unit box b = 5 nm. When the unit box is small enough, the periphery of the Pd nanocrystals cut in a unit can be regarded as a straight transversal, and the expectation value L is $\frac{\pi}{4}b$.

Through a counting process, we got the number of boxes which were fully covered with the Pd nanocrystal (S), and the number of boxes which were partially covered or involved a section of the periphery (P). In the zones involved with several layers, repeat counting was utilized.

The area of planar surface $A_{\{111\}} = Sb^2 + \frac{1}{2}Pb^2$.

The area of edges $A_{edge} = (h\gamma)P\frac{\pi}{4}b$. h is the average thickness of the Pd nanocrystals, which is obtained statistically from TEM images. γ is the shape factor of the edges in nanostructures, which is used to revise the thickness as a multiplier for A_{edge}. γ can be calculated as: $\gamma = \frac{2\cdot\sqrt{(\frac{h}{2})^2 + d^2}}{h}$, where the cross section of edges are regarded as isosceles triangles and d is the altitude on the base. The average value of d can be derived from the atomic HAADF-STEM images of the edges, the contrast profiles in Figure 2 and Figure S7-9 are examples.

The total surface area is $A = A_{\{111\}} + A_{\text{edge}}$. Then we can get the percentages of edge area in total surface area.

In addition, we can derive the percentage of surface atoms in a Pd nanocrystal from ECSA: $\frac{N_s}{N} = \frac{ECSA \cdot \rho_{111}}{N_A}$. N_s and N are the surficial and total atomic numbers; $\rho_{111} = \frac{4}{\sqrt{3}a^2}$ is the atomic density of the Pd {111} planes which are dominant in the nanocrystals; N_A is Avogadro's number and a is the lattice constant of *fcc* Pd. The ratio of Pd atoms at edge states related to total atoms is approximately denoted as $\frac{N_{edge}}{N} = \frac{N_s}{N} \cdot \frac{A_{edge}}{A}$.

As for the area specific activity of a Pd catalyst we prepared, we can denote it as: $i_s = \sum i_k \cdot \frac{A_k}{A}$ (k = 0, 1, 2, 3, 4). i_0 and $\frac{A_0}{A}$ denote the specific activity and percentage of the Pd {111} planes while i_k and $\frac{A_k}{A}$ (k = 1, 2, 3, 4) denote the specific activity and percentage of edge area of different edge states in Pd FNSs, PNSs NBs and 2D NFs. According to a series of morphological and structural characterizations, we found out the crystal structures and chemical states of edges in Pd FNSs (edge 1) and Pd 2D NFs (edge 4) are very similar. Make $i_1 = i_4$, we can solve all equations and obtain the approximate results including the ratios of $\frac{i_k}{i_0}$ and the activity percentages contributed by edge sties.

Supplemental data

Supplemental tables.

Table S1. The quantitative analysis on the characteristic statistical parameters of the Pd nanostructures which describe the features of shape and edge sites.

Samples	Thickness [nm]	D[a)]	γ[b)]	A_{edge}/A [%][c)]	N_S/N [%][d)]	N_{edge}/N [%][e)]
Pd FNSs	1.2	2.1	1.9	8.37	19.24	1.61
Pd PNSs	1.5	1.8	3.5	30.12	12.87	3.88
Pd NBs[*]	3.9	1.5[f)]	1.4	37.01	4.48	1.66
Pd 2D NFs	2.0	1.6	1.8	28.67	8.77	2.51

a) ***D*** denotes the fractal dimensions of Pd 2D nanostructures.

b) γ denotes the corresponding shape factors of the edges.

c) A_{edge} and ***A*** denote the area of the edges and total surface areas.

d, e) N_S, N_{edge}, and ***N*** denote the numbers of surface atoms, edge atoms and total atoms of a Pd 2D nanostructure.

f) The ***D*** of Pd NBs is calculated for relatively isolated nanocrystals while 3D network superstructure of these nanobelts may form via aggregation.

Table S2. The results of the electrochemical measurements of Pd catalysts.

Samples	*ECSA* [m^2/g]	i_s [mA/cm^2]	i_m [mA/mg]	$i_{m\text{-}500}$ [mA/mg][a)]	$i_{m\text{-}500}/i_m$ [%]
Pd black	16.1	1.91	308	11	3.8
Pd FNSs	71.3	1.10	783	259	33.1
Pd PNSs	47.7	2.08	989	114	11.5
Pd NBs	16.6	2.68	445	275	61.8
Pd 2D NFs	32.5	2.89	940	432	46.0

a) $i_{m\text{-}500}$ is the mass activity after 500 cycles in FAOR measurements.

Supplemental figures.

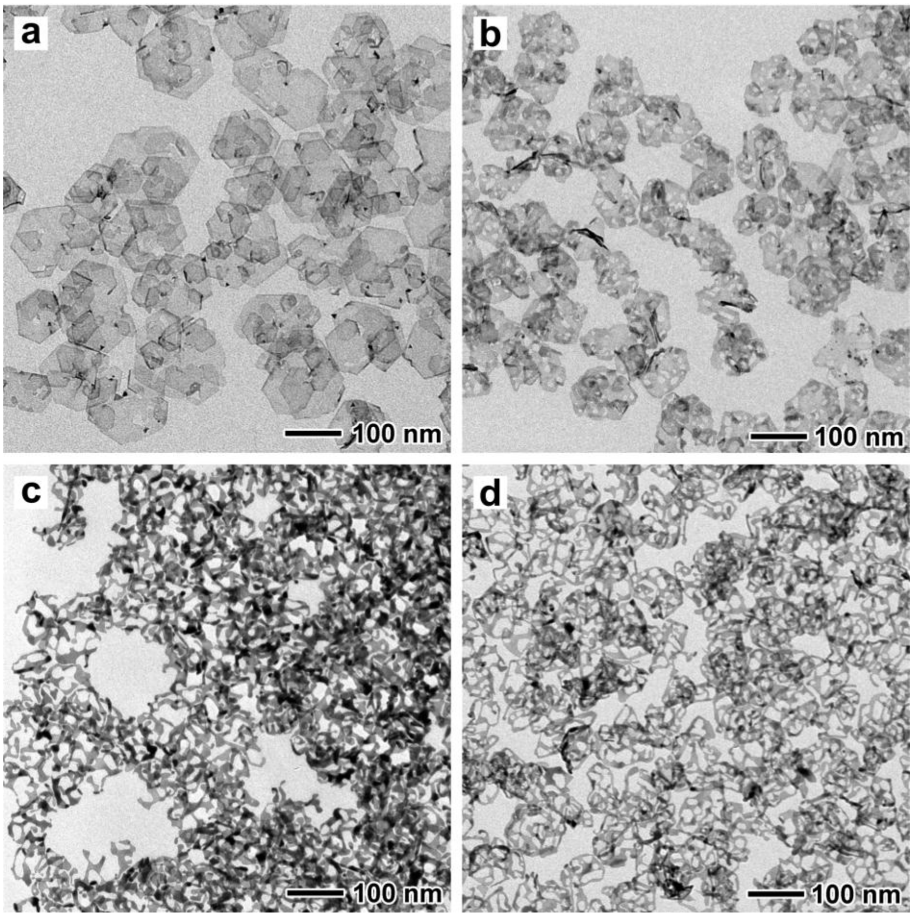

Figure S1. TEM images for the (a) Pd FNSs, (b) Pd PNSs, (c) Pd NBs, and (d) Pd 2D NFs at lower magnification.

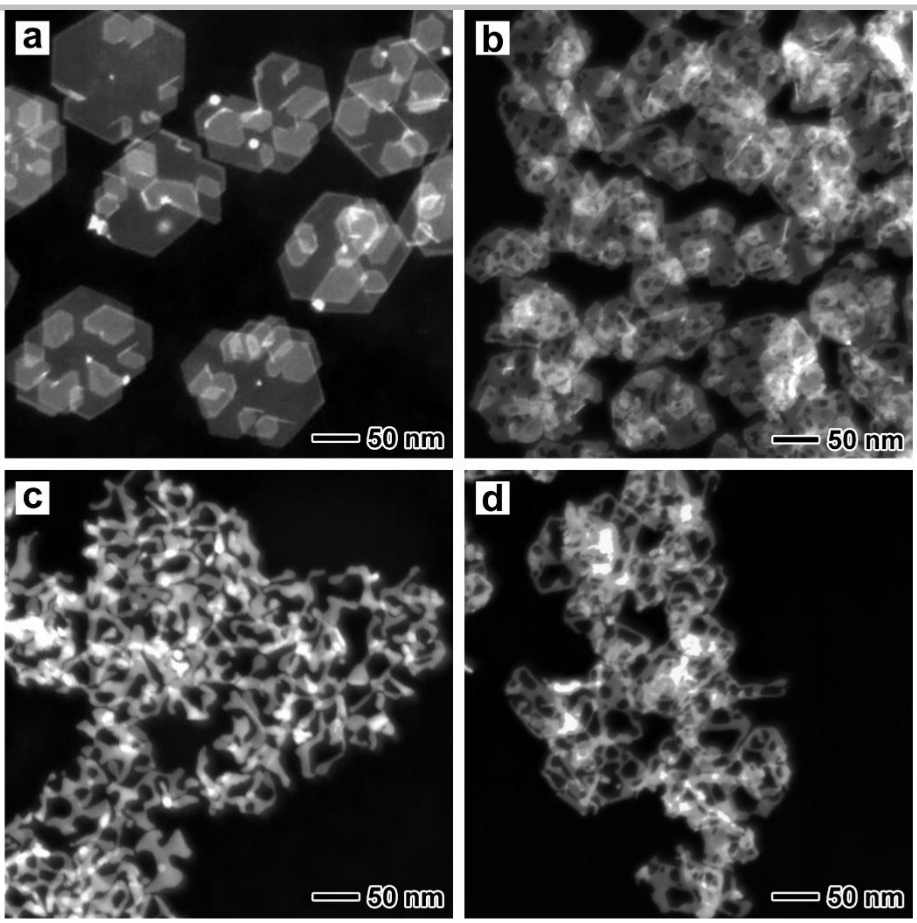

Figure S2. HAADF-STEM images for the (a) Pd FNSs, (b) Pd PNSs, (c) Pd NBs, and (d) Pd 2D NFs.

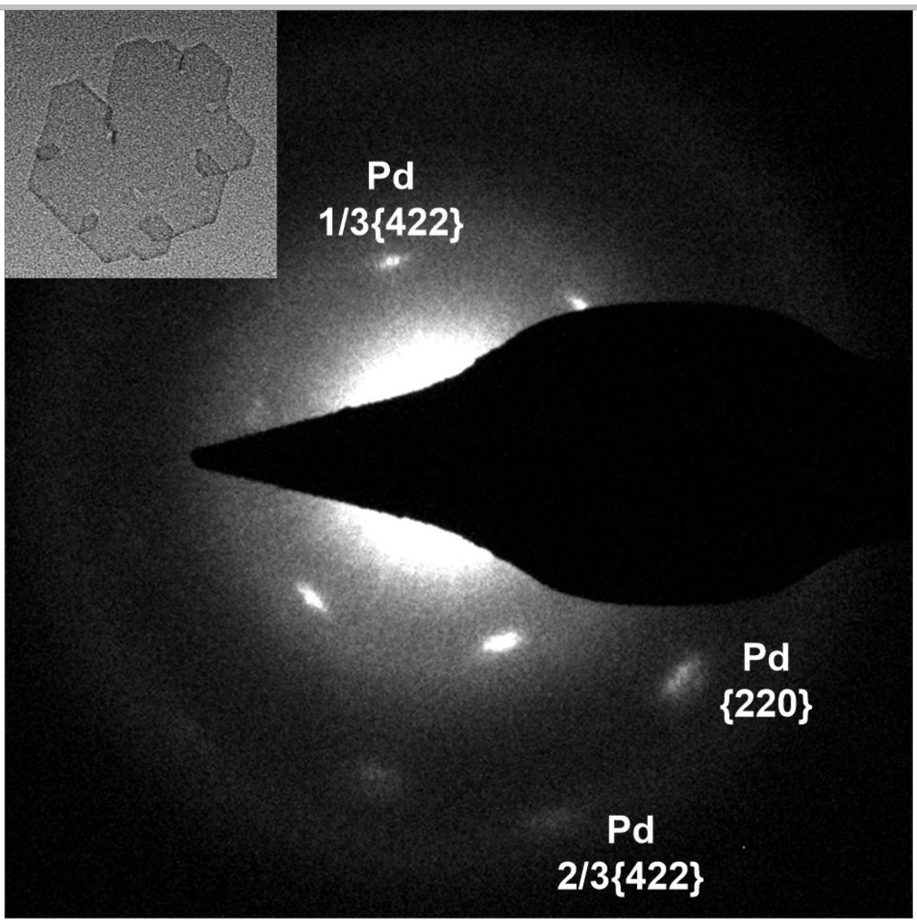

Figure S3. SAED pattern for a single Pd FNS measured along the <111> direction. The inset is the TEM image of the corresponding area.

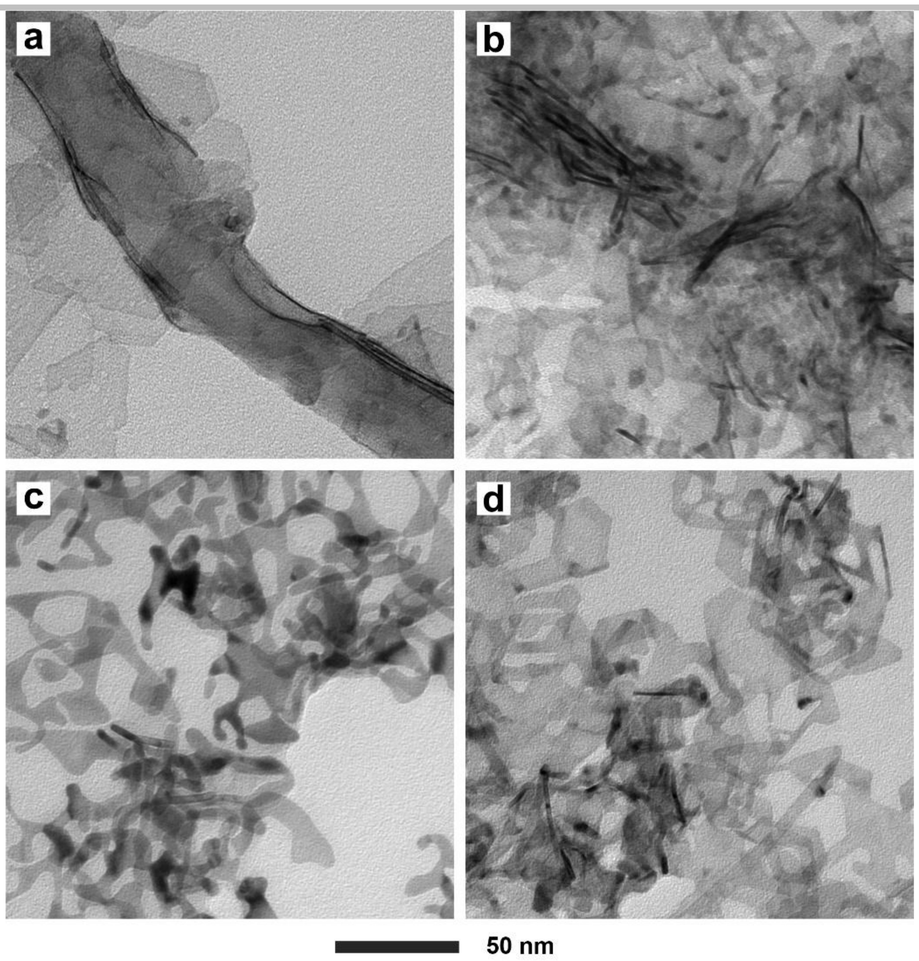

Figure S4. Representative TEM images for the (a) Pd FNSs vertically attached on the carbon nanotubes, (b) Pd PNSs, (c) Pd NBs, and (d) Pd 2D NFs vertically assembled for the statistics of average thickness.

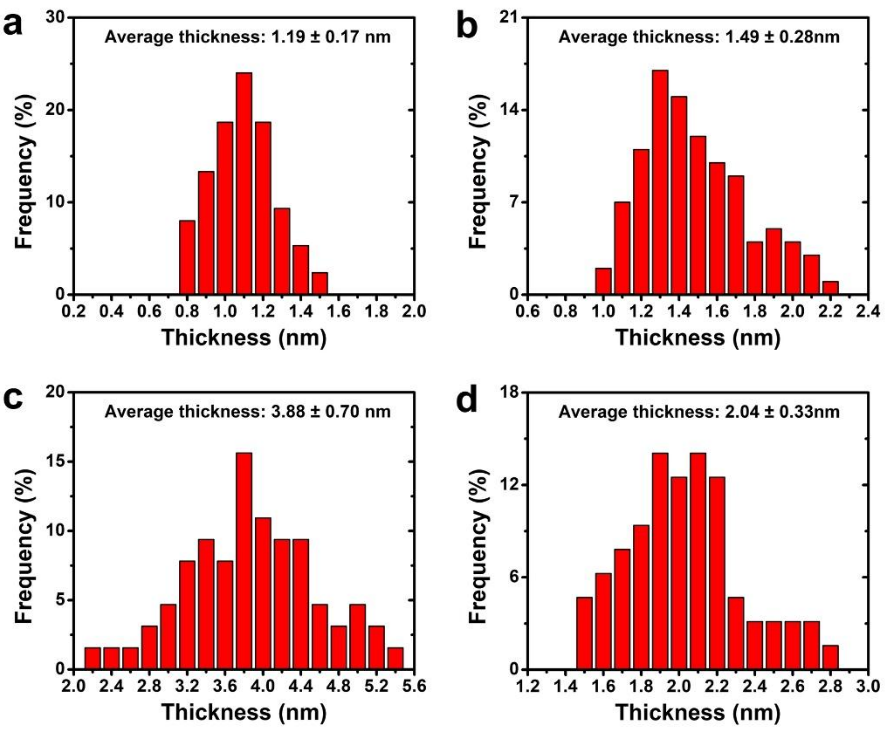

Figure S5. The thickness distribution of the (a) Pd FNSs, (b) Pd PNSs, (c) Pd NBs, and (d) Pd 2D NFs.

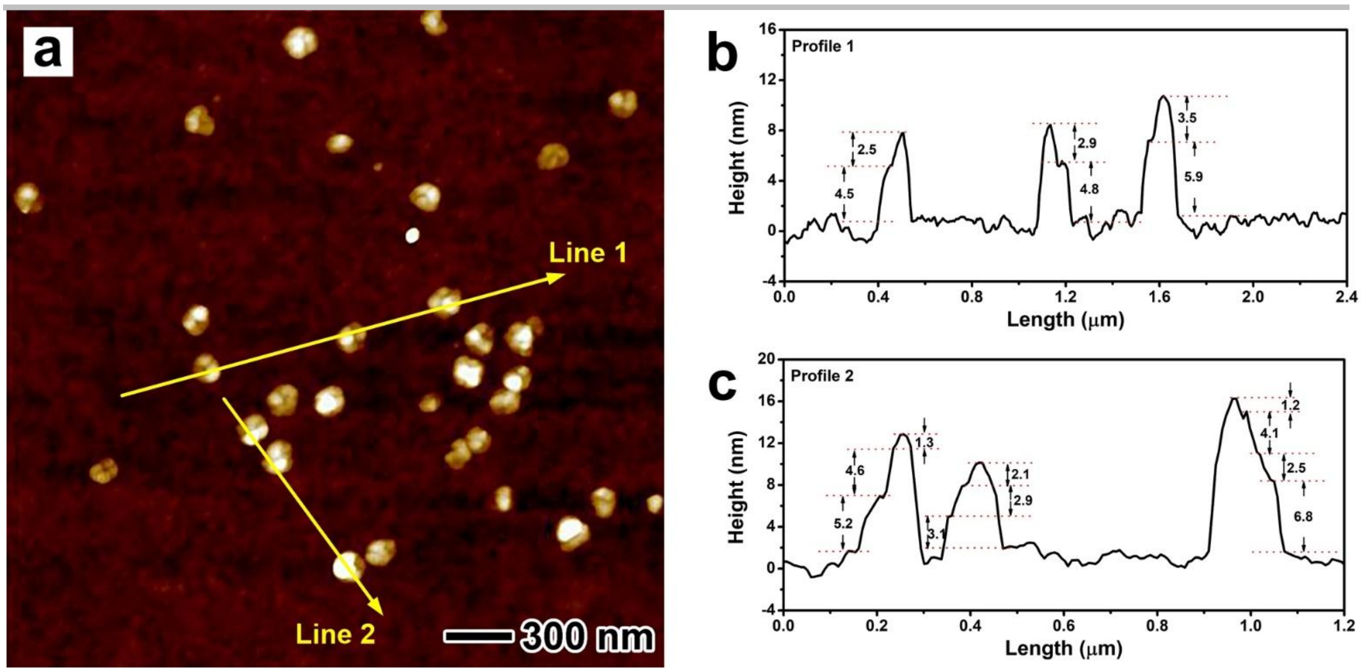

Figure S6. (a) AFM height image for the Pd FNSs on the Si/SiO_2 wafer and (b, c) section height profiles measured along the corresponding lines in (a).

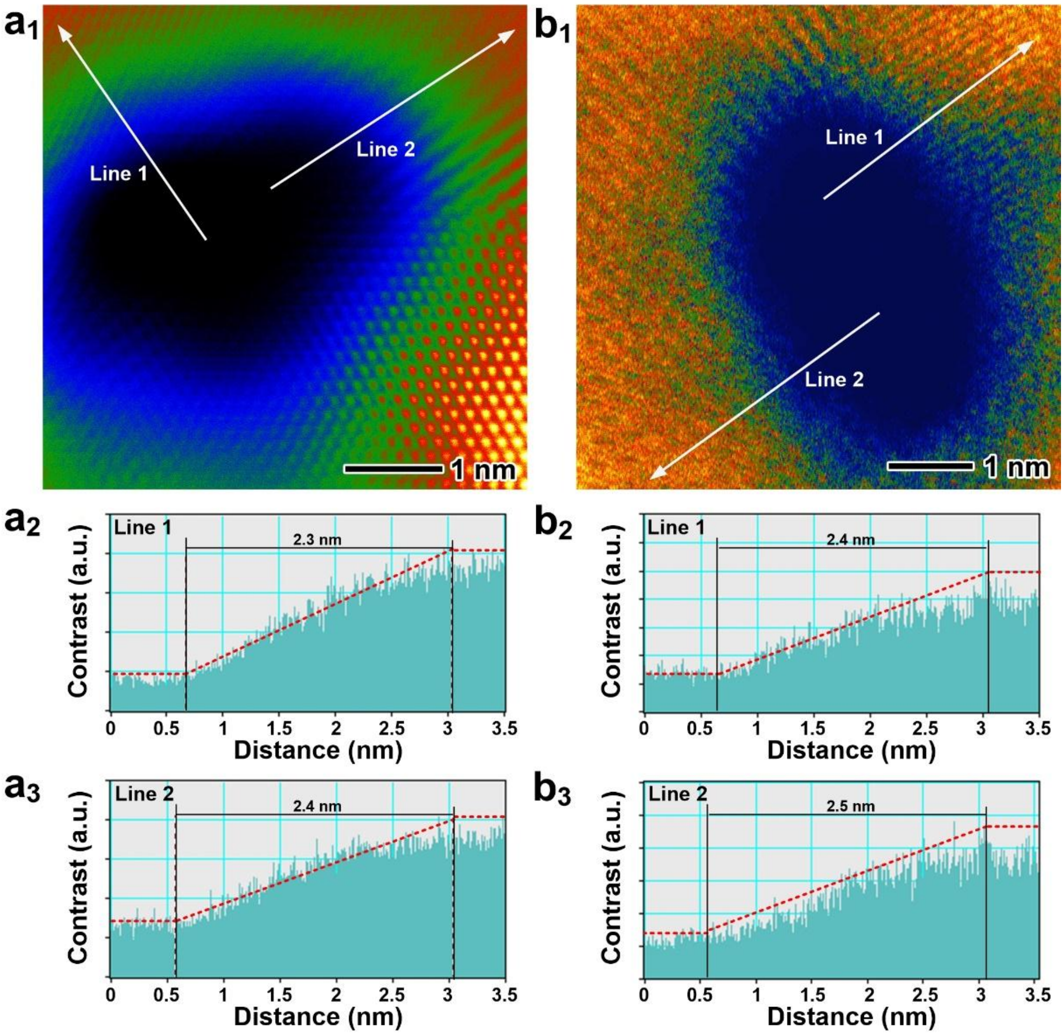

Figure S7. (a_1, b_1) Atomic resolution aberration-corrected HAADF-STEM images and (a_2-a_3, b_2-b_3) contrast profiles measured along the arrowed lines of edges with different orientations in the Pd PNSs. False color was applied to enhance the contrast.

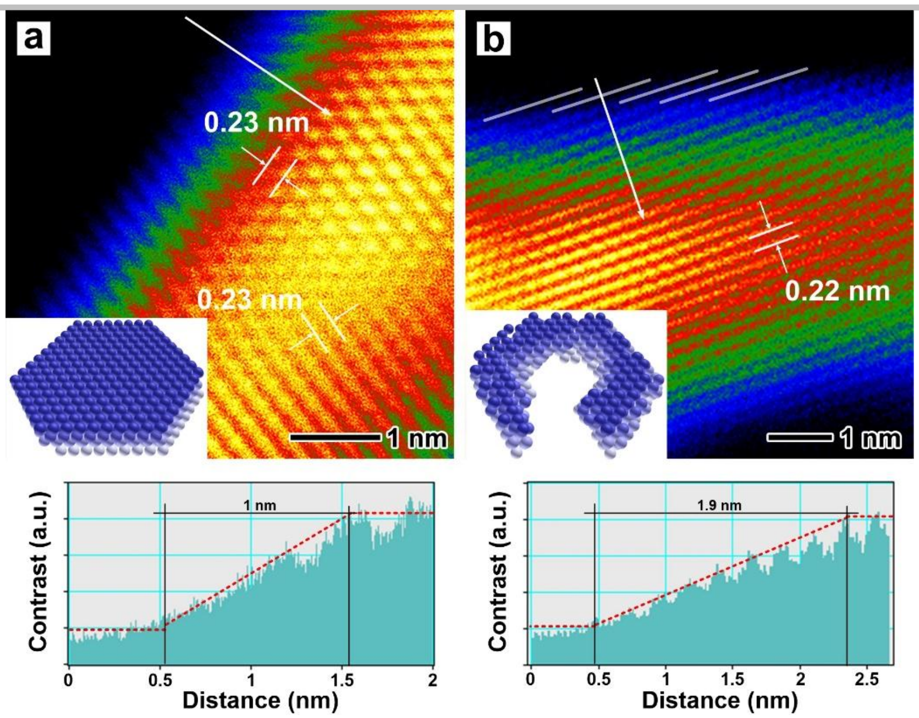

Figure S8. Atomic resolution aberration-corrected HAADF-STEM images and contrast profiles measured along the arrowed lines of outer edges in the a) Pd FNSs and b) Pd NBs. False color was applied to enhance the contrast. The insets show the corresponding atomic models.

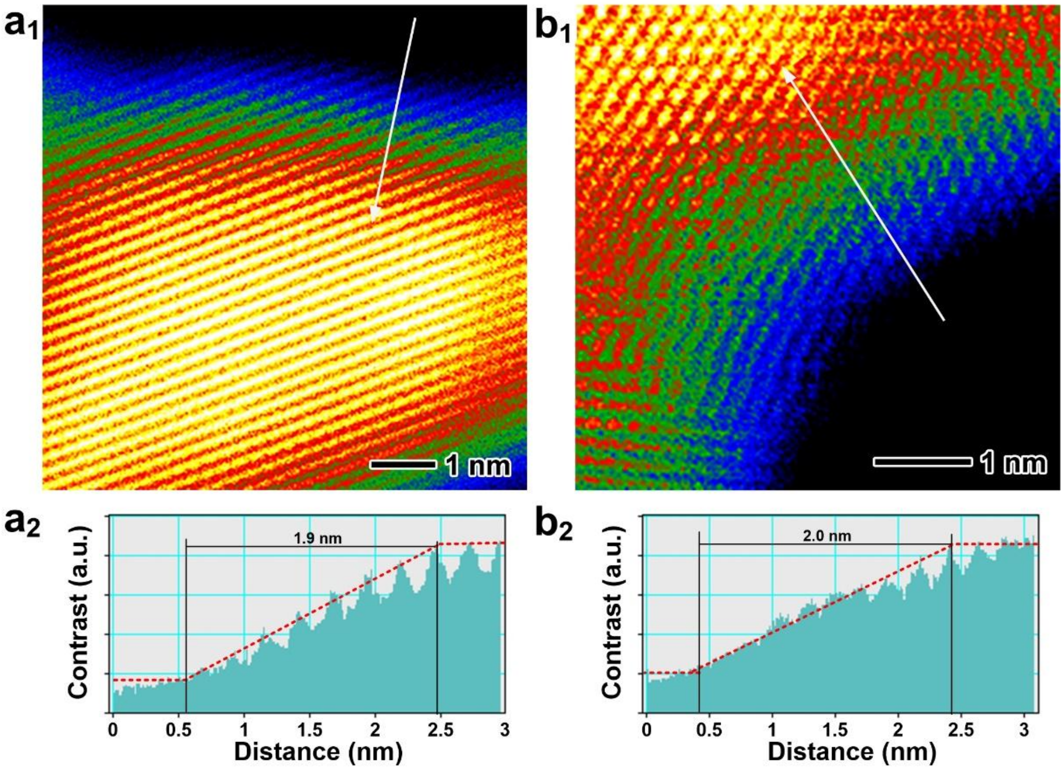

Figure S9. (a_1, b_1) Atomic resolution aberration-corrected HAADF-STEM images and (a_2, b_2) contrast profiles measured along the arrowed lines of edges with different orientations in the Pd NBs. False color was applied to enhance the contrast.

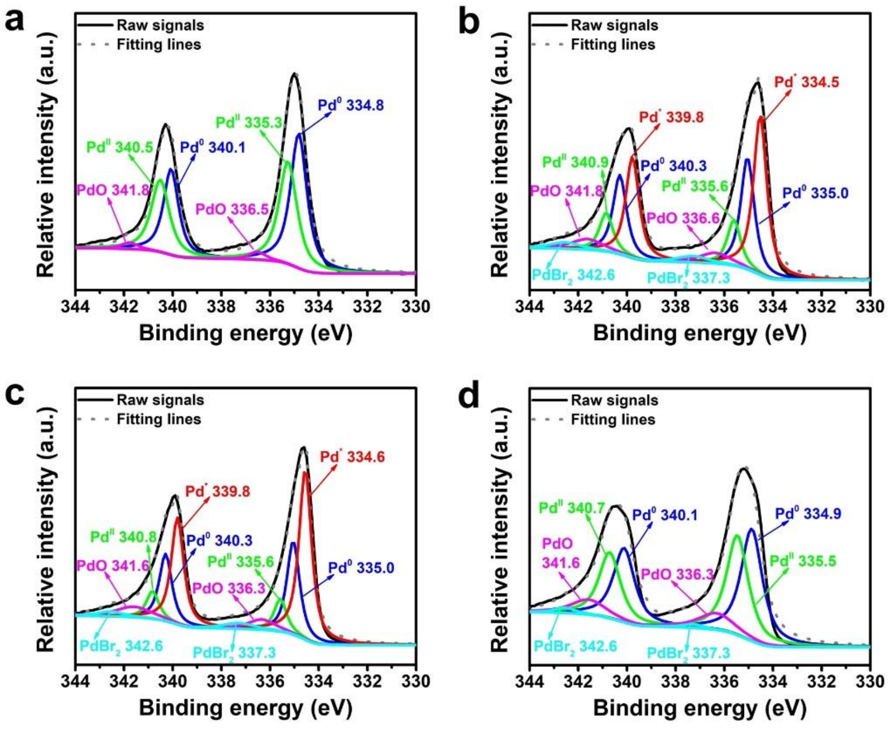

Figure S10. XPS spectra of the 3d peaks for the (a) Pd FNSs, (b) PNSs, (c) NBs and (d) 2D NFs.

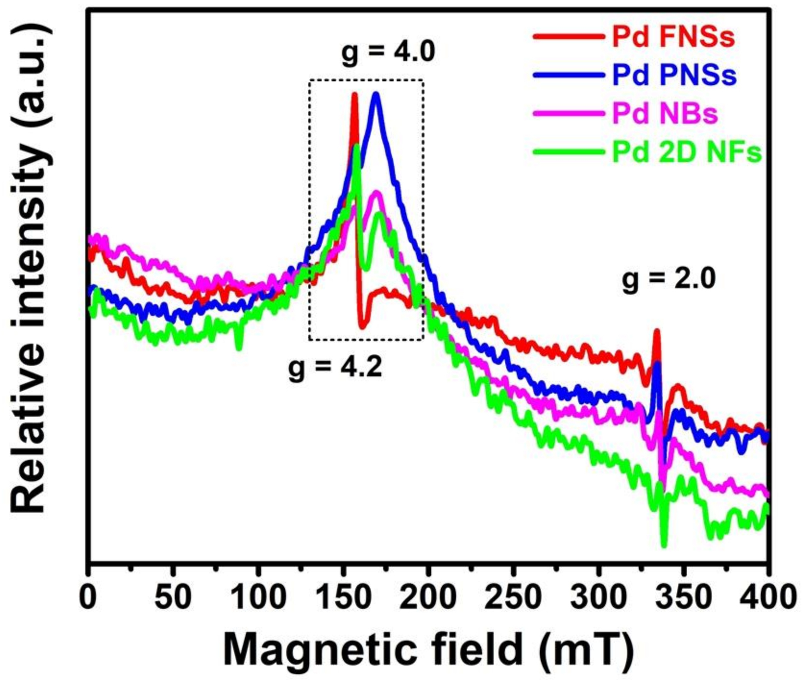

Figure S11. EPR spectra for the Pd FNSs, PNSs, NBs and 2D NFs.

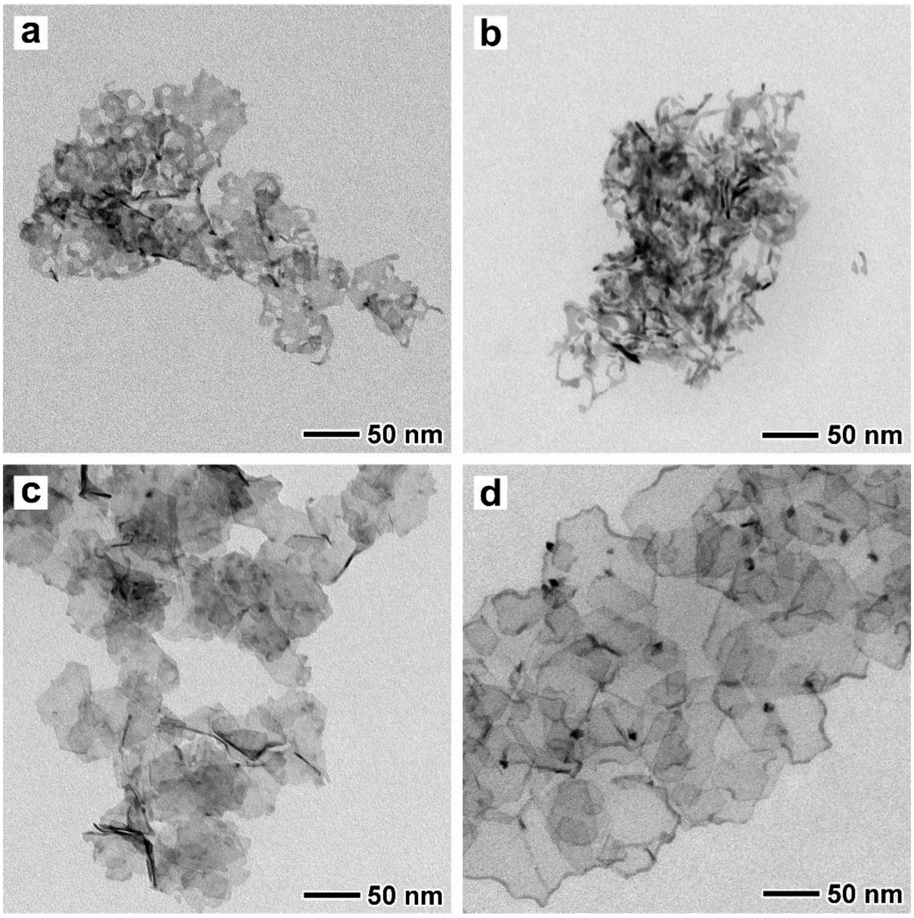

Figure S12. Representative TEM images for the Pd nanosheets prepared by standard procedures of Pd PNSs (a) without the addition of AA and NaAc, (b) with the addition of 10 μL acetic acid instead of AA, (c) with the addition of 10 mg NaAc and without the addition of AA, (d) with the addition of 10 mg NaAc.

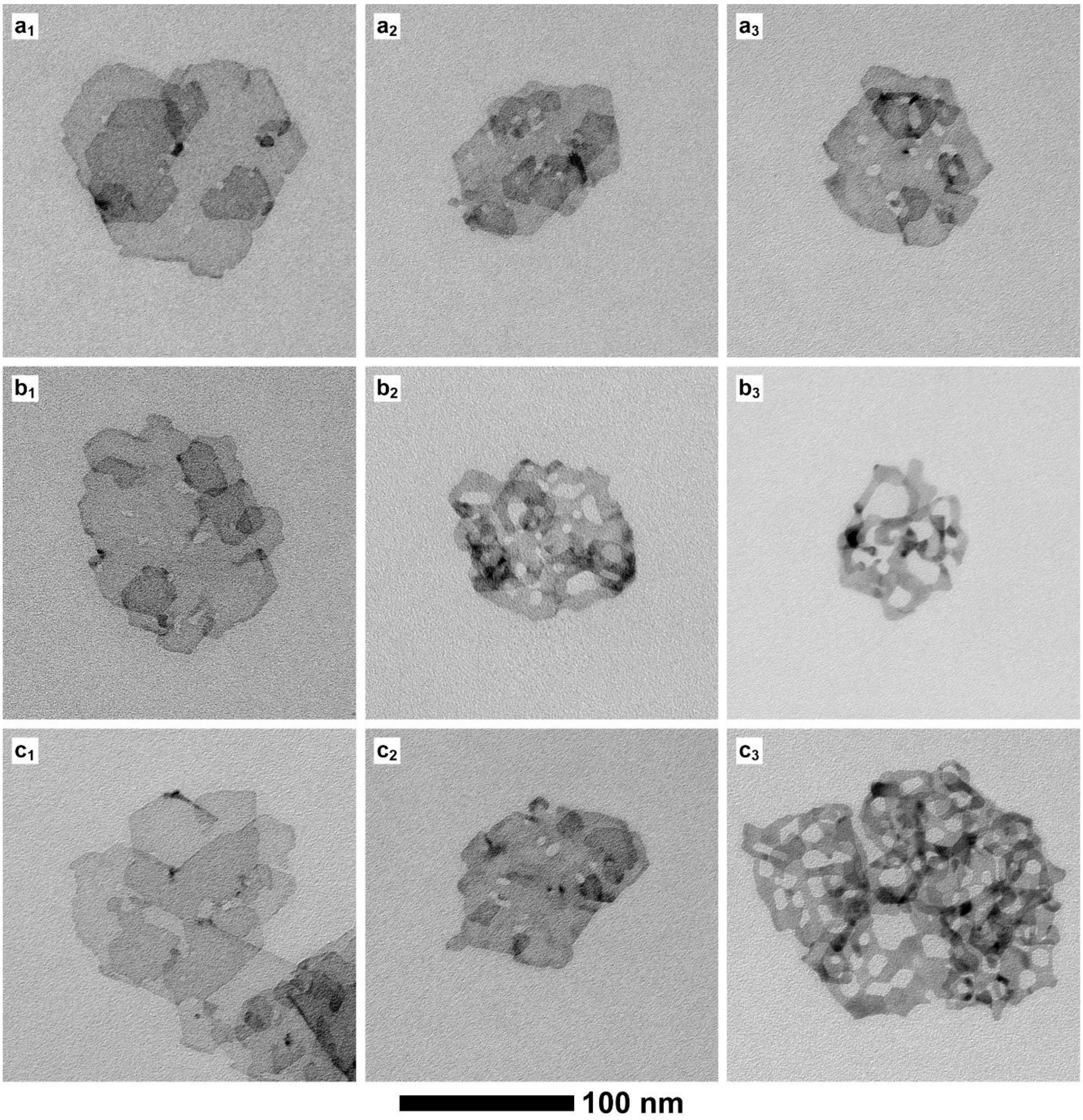

Figure S13. Representative TEM images for the (a_1-a_3) Pd PNSs, (b_1-b_3) Pd NBs, and (c_1-c_3) Pd 2D NFs obtained at different synthetic reaction duration of 15, 30, and 60 min.

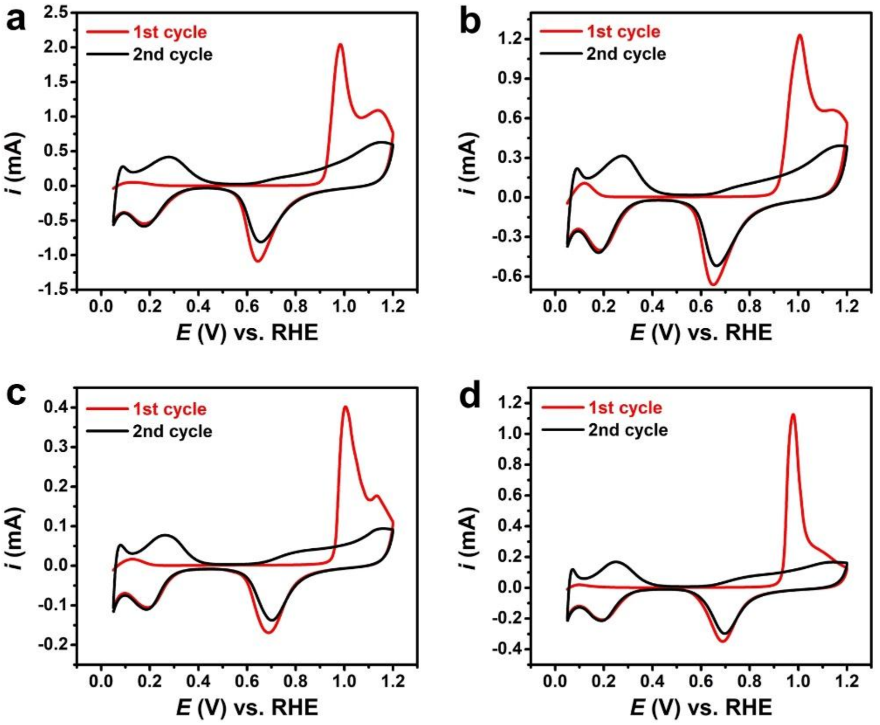

Figure S14. CO stripping curves for the (a) Pd FNSs, (b) Pd PNSs, (c) Pd NBs, and (d) Pd 2D NFs in 0.1 M $HClO_4$ solution at a scan rate of 50 mV/s.

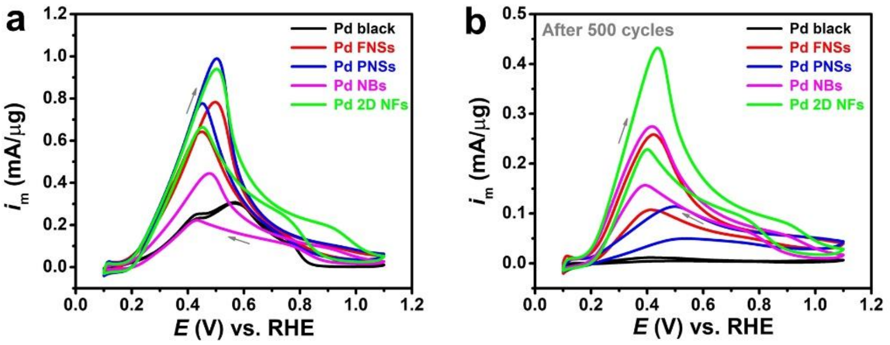

Figure S15. Cyclic voltammograms (CV) for the (a) as-prepared Pd black, FNSs, PNSs, NBs and 2D NFs and (b) samples after 500 cycles. The CV curves were measured in a mixed solution containing 0.5 M H_2SO_4 and 0.5 M formic acid at a scan rate of 50 mV/s for FAOR normalized by Pd mass.

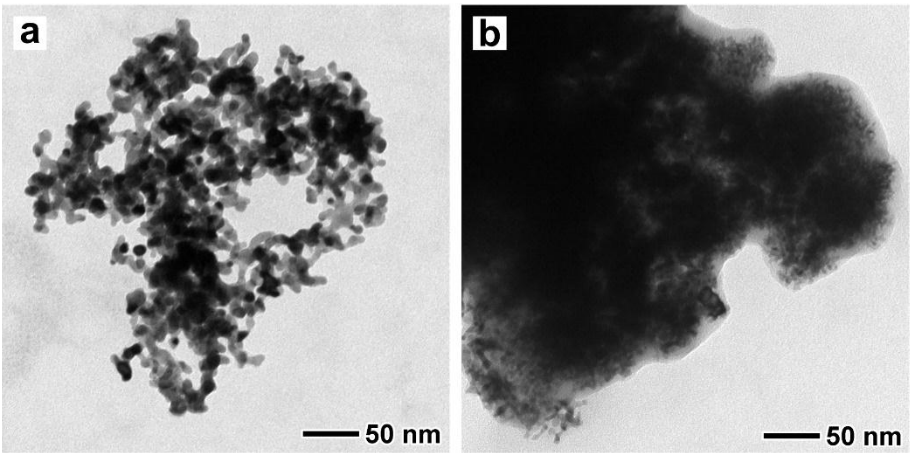

Figure S16. Representative TEM images of commercial Pd black (a) before and (b) after electrochemical durability tests.

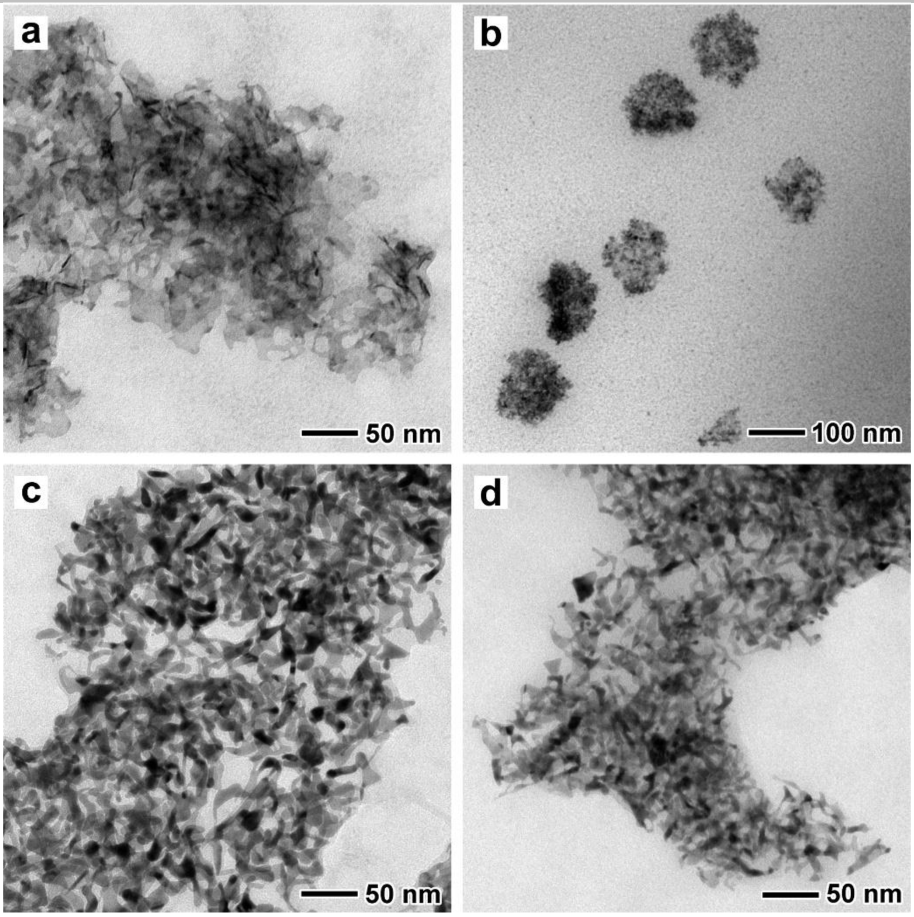

Figure S17. Representative TEM images for the (a) Pd FNSs, (b) Pd PNSs, (c) Pd NBs, and (d) Pd 2D NFs after electrochemical durability tests.

References

[1] B. B. Mandelbrot, *The Fractal Geometry of Nature*, W. H. Freeman, **1982**.

[2] J. Shang, Y. Wang, M. Chen, J. Dai, X. Zhou, J. Kuttner, G. Hilt, X. Shao, J. M. Gottfried, K. Wu, *Nat. Chem.* **2015**, *7*, 389-393.

[3] P. Pfeifer, D. Avnir, *J. Chem. Phys.* **1983**, *79*, 3558-3565.
